# Review of applications and challenges of quantitative systems pharmacology modeling and machine learning for heart failure

**DOI:** 10.1007/s10928-021-09785-6

**Published:** 2021-10-12

**Authors:** Limei Cheng, Yuchi Qiu, Brian J. Schmidt, Guo-Wei Wei

**Affiliations:** 1 Quantitative Systems Pharmacology and Physiologically Based Pharmacokinetics, Bristol Myers Squibb, Princeton, NJ 08536 USA; 2 Department of Mathematics, Michigan State University, East Lansing, MI 48824 USA; 3 Department of Electrical and Computer Engineering, Michigan State University, East Lansing, MI 48824 USA; 4 Department of Biochemistry and Molecular Biology, Michigan State University, East Lansing, MI 48824 USA

**Keywords:** Quantitative systems pharmacology, QSP modeling, Machine learning, Heart failure, Physiological modeling, Modeling and simulation

## Abstract

Quantitative systems pharmacology (QSP) is an important approach in pharmaceutical research and development that facilitates in silico generation of quantitative mechanistic hypotheses and enables in silico trials. As demonstrated by applications from numerous industry groups and interest from regulatory authorities, QSP is becoming an increasingly critical component in clinical drug development. With rapidly evolving computational tools and methods, QSP modeling has achieved important progress in pharmaceutical research and development, including for heart failure (HF). However, various challenges exist in the QSP modeling and clinical characterization of HF. Machine/deep learning (ML/DL) methods have had success in a wide variety of fields and disciplines. They provide data-driven approaches in HF diagnosis and modeling, and offer a novel strategy to inform QSP model development and calibration. The combination of ML/DL and QSP modeling becomes an emergent direction in the understanding of HF and clinical development new therapies. In this work, we review the current status and achievement in QSP and ML/DL for HF, and discuss remaining challenges and future perspectives in the field.

## Introduction

### Heart failure

Heart failure (HF) is generally described as a condition where the blood output of the heart is not sufficient to meet metabolic demands of the tissues [1]. However, a variety of dysfunctions comprise heart failure, heart failure can be a challenging syndrome to diagnose clinically, and compensatory cardiac mechanisms can sometimes mask an underlying disease state. A critical functional quantity used in the characterization of heart failure is the ejection fraction (EF), the ratio between the blood volume ejected and total filled volume. EF is usually reported for the cardiac chamber responsible for systemic delivery of arterial blood through the aorta, the left ventricle ejection fraction (LVEF). The two most commonly diagnosed types of heart failure are heart failure with reduced ejection fraction (HFrEF) and heart failure with preserved ejection fraction (HFpEF). Normally, the EF of a healthy person is 55–70%, and when EF drops below 40 %, it is characterized as HFrEF. Intermediate ejection fractions of 40–55% are often considered to be abnormal [2]. For HFpEF patients both the blood ejected volume and the total filled blood volume are reduced, which results in a near-normal, preserved EF.

Heart failure can have a number of etiologies, but generally progresses from an initial cardiac injury through a remodeling stage that ultimately results in cardiac dysfunction [3]. The causes of cardiac injury can include myocardial disease, pericardial or endocardial abnormalities, valve diseases such as regurgitation and stenosis, arrhythmia, pressure overload, or volume overload [4]. The most common cause is cardiac injury is myocardial ischemia, which usually involves blockage of the coronary arteries from coronary artery disease (CAD) [4]. Remodeling is driven by cellular processes including cardiomyocyte hypertrophy, proliferation of fibroblasts, extracellular matrix changes including an initiation of fibrosis, apoptosis and necrosis of myocytes, and inflammation [4]. Hypertrophy can be stimulated by reductions in blood flow to the heart, which results in increase oxygen demand. In addition to increased demand for oxygen with additional muscle tissue, the increased wall stiffness can serve as a barrier to sufficient coronary blood flow. This positive cycle causes cardiac remodeling and is often associated with worsening in HFpEF. Cardiac myocyte apoptosis and necrosis results in weakened heart muscle, reduced force of contraction, reduced stroke volume, and ultimately produces remodeling with progressively thinner walls and larger ventricle chambers in HFrEF [5].

Increases in fluid retention and congestion are additional hallmarks of heart failure. When the heart does not contract properly in heart failure, less blood passes through the heart and lungs. The kidney compensates for the response in arterial underfilling as triggered by baroreceptor activation as well as neurohormonal stimulation, increasing sodium and water retention. Gradually, the interstitial fluid and plasma volume expand [6]. This results in congestion in the lungs and body and fluid retention in tissues and organs. In addition to the loss of cardiac functionality, heart failure therefore also progressively results in shortness of breath, swelling, and the loss of the ability to exercise.

No single mechanism can account for the heterogenous clinical syndromes and conditions of heart failure. Thus, multiple strategies are needed to assess the mechanisms, complex systems, describe the clinical syndromes, and assess the likelihood of the many alternate causes of heart failure [7].

### Introduction of quantitative systems pharmacology

Mathematical biology and pharmacology models are increasingly used in preclinical and clinical drug development. Quantitative systems pharmacology (QSP) is an emerging interdisciplinary field that integrates systems biology and pharmacometrics, with an emphasis on dynamic modeling, to quantitatively predict the effects of clinical interventions and their combinations under a variety of genetic, biochemical, biophysical, biomechanical, and physiological conditions. Sometimes these mechanistic underpinnings are also mapped to influential demographic considerations such as gender, race, and age [8, 9]. Recently, machine learning (ML) approaches to inform QSP have been of increasing interest and utility. QSP models are developed by incorporating underlying disease and therapeutic mechanisms, and are leveraged to solve complex problems, improve decisions, and reduce costs in drug development. They are applied from early drug design and discovery to late-stage clinical development. A fast-growing interest in application of QSP to dosing and trial decision-making for clinical development has made QSP a well-recognized tool in drug development. QSP has supported clinical development in many therapeutic areas and provided unexpected insights [10]. The further integration, calibration, and validation with patient data, particularly those from clinical trials, enables QSP to provide mechanical and actionable predictions. QSP modeling has become an indispensable tool that has also created interest from regulatory agencies such as the US Food and Drug Administration (FDA) and European Medicines Agency (EMA) for new drug development [10, 11]. As such, QSP will have a substantial impact on human healthcare and the pharmaceutical industry in the future.

### Overview of machine learning and deep learning

Machine learning (ML), including deep learning, has had a growing and substantial impact on science, engineering, technology, and industry in the past decade [12, 13]. It is a branch of artificial intelligence and computer science, which focuses on the use of data and algorithms to imitate the way that humans learn, gradually improving its accuracy [14]. Supervised learning and unsupervised learning are two major categories that have a wide clinical application on HF [15], although semi-supervised learning and self-supervised learning are potentially useful. In this section, we give a brief introduction to these areas of machine learning algorithms.

#### Supervised learning

The primary difference between supervised learning and unsupervised learning is whether the training data is “labeled.” Supervised learning uses labeled datasets to train the ML model. Supervised learning consists of two major types of tasks, regression and classification, where a regression task predicts a continuous quantity and a classification task generally predicts a category. Linear regression is one basic regression algorithm that uses a linear approach to model the relationship between a scalar response and one or more explanatory variables (i.e. predictors) [16]. Logistic regression is used to model the probability of a certain class or event existing, which is mainly intended for classification problems [17]. Support vector machine (SVM) is a classification method that develops an optimal hyperplane that maximally separates high dimensional data points classified into two categories. Although the hyperplane is a linear divider, nonlinear classification problems can be tackled with a kernel function. New examples are then mapped into that same space and predicted to belong to a category based on which side of the line they fall [18]. SVM has also been adapted for regression problems, support vector regression (SVR). Another classification method is the decision tree, a flowchart-like structure with many binary decision points, or nodes. Leaf nodes are the end points in a decision tree structure, and each leaf node assigns a class label. The paths from the root to the leaf represent classification rules [19]. Decision trees have been noted to be prone to overfitting, whereby they may fit the data very well, including spurious features and noise. An ensemble method, i.e. the random forest method, proceeds by constructing multiple decision trees at training time and averaging predictions of the individual trees to mitigate overfitting issues [20]. Many additional ensemble learning methods have been developed, such as gradient boosting trees and XGBoost [21].

Deep learning has become more popular in supervised learning because of its capabilities for universal approximation and availability of increasingly powerful high-performing computational tools. One popular deep learning algorithm is the artificial neural network (ANN), a network consisting of connected units or nodes [22]. Each node produces a single output value based on a weighted sum of the inputs plus a bias term. Intermediate nodes between the input and output nodes are often called hidden nodes, and there may be multiple layers of intervening nodes. To prevent overfitting and reduce the dimensionality of the model in the neural network, convolution neural networks (CNN) introduced convolutional layers, which perform additional matrix multiplication operations on the input. CNNs are particularly designed for learning image data [23]. Recurrent neural networks (RNN) include additional recurrence relationships that rely on previous network time points that are not included in ANNs but are needed to help predict sequences, such as letters in sentences and words. In natural language processing, many RNN architectures were introduced to make the model scalable to sequence data with various lengths, such as long short-term memory (LSTM) [24], gated recurrent unit (GRU) [25] and transformer [26]. ML models can simultaneously take multiple features or biomarkers as input data, and meanwhile predict multiple clinical events or outputs. The existence of multiple output data enables the use of the so-called to multitask deep learning (MDL) method. This approach, also called transfer learning, can take the advantage of a large data set to help improve the prediction accuracy of small data sets.

#### Unsupervised learning

Another type of machine learning task is unsupervised learning, where the data have no labels. Two of the main methods in unsupervised learning are dimension reduction and clustering. Dimension reduction transforms high-dimensional data into a low-dimensional space retaining the most meaningful properties. Clustering divides data into multiple groups with similar internal characteristics. Dimension reduction methods are common in fields that deal with high-dimensional data for noise reduction, data visualization, and cluster analysis [27]. Linear dimension reduction, such as principal component analysis (PCA), performs a linear mapping of the data to eigenspace in lower dimension, maximally preserving the variability in the original data while using minimal dimensions in the transformed space [28]. Other nonlinear dimension reduction methods, such as non-negative matrix factorization (NMF) [29], T-distributed stochastic neighbor embedding (t-SNE) [30], autoencoder [31], and uniform manifold approximation and projection (UMAP) [32], were widely used to data with different structures [33]. Clustering is the task of grouping a set of objects in such a way that objects in the same group are more similar to each other than to those in other groups. Distance-based clustering [34, 35], hierarchical clustering [36], community-based clustering [37, 38], density-based clustering [39], soft clustering [40, 41], and graph-based clustering [42] were widely applied to transcriptomic data analysis [43], pattern recognition [44], image processing [45] as well as heart failure [46] to reveal data internal characteristics.

## QSP modeling for cardiovascular diseases and heart failure

### Applications of QSP modeling for cardiovascular diseases and heart failure

For pharmaceutical discovery and development, one crucial question that must be addressed is whether the potential treatment is safe and efficacious. Mechanistic systems modeling approaches can provide insights and guide target identification and drug evaluation throughout the drug development process [47]. QSP models can improve understanding of heart failure mechanisms, provide safety and efficacy assessments, and assist in the design and planning of clinical trials.

QSP modeling has been applied in the pharmaceutical industry to cardiovascular applications. As one example, the Cardiovascular Physiolab® platform models the biology and pathophysiology of cardiovascular disease and was applied to explore the progression of atherosclerosis and response to therapies [48]. One striking prediction from the model was that although cholesterol ester transfer protein (CETP) inhibition can modulate the circulating lipoprotein profile, the known mechanisms for clinically explored CETP inhibitors generally would not effectively remove cholesterol from plaque to facilitate improved cardiovascular outcomes. That is, the model both provided a quantitative hypothesis for the lack of impact of torcetrapib on plaque outcomes and further suggested a class effect posing a challenge to the clinical development of CETP inhibitors that would extend beyond torcetrapib. The mechanistic insight and predictions were made public prior to the readouts of multiple phase 3 trials for CETP inhibitors with outcomes in agreement with the insights provided by the mechanistic modeling work [47–49].

For heart failure patients, reduced cardiac output and arterial filling pressure leads to congestion in the lungs and body, which causes short breath and fluid retention, contributing to further volume overload. As one example of mechanistic modeling applied to heart failure, cardiac and renal interactions in HFrEF were modeled by Yu et al. to incorporate the underlying mechanisms of edema and investigate the effects of renally-targeted therapies on HFrEF [50]. The model investigated the poorly understood effects of sodium-glucose co-transporter 2 inhibitors (SGLT2i) on HF. Simulated virtual diabetic and non-diabetic HF patients and simulated clinical trials were used to evaluate the effect of SGLT2i on cardiac functions, blood volume, congestion, and edema in HFrEF patients. Yu et al. proposed that HFrEF improves with SGLT2 inhibition due by reducing cardiac preload and relieves congestion by reducing the interstitial fluid accumulation.

Myocardial energetics also change with decompensated heart failure. However, it is unknown how mechanical function and changes in myocardial energetics interact with each other in HF. Tewari et al. developed an integrated model with mitochondrial ATP energetics, calcium-dependent actin-myosin cross-bridge cycling, and systemic resistance to address this question [51]. The model was applied to investigate the efficacy of drugs, including omecamtive mecarbil, on cross-bridge cycling kinetics and resulting oxygen demands in decompensated heart failure. Tewari et al. used the computational analysis to propose how metabolic changes can account for the systolic dysfunction in heart failure.

QSP modeling in HF often uses data from literature, nonclinical studies, and available prior clinical data to inform model develop and assist clinical studies and development. There are many open questions about the development of therapeutics for heart failure that would benefit from a QSP modeling approach. QSP can address early assessments of clinical efficacy, assessments of the optimal dose level, dosing regimen optimization, combination with other therapeutics including the standard of care, and identification of characteristics of responders in heterogenous patient populations. QSP models can also be updated with emerging clinical data. When the primary outcome fails in a clinical trial, QSP can be applied to re-assess whether there is a strong biologic rationale that favors the treatment and whether the failure was due to the trial population [52].

**Fig. 1:**
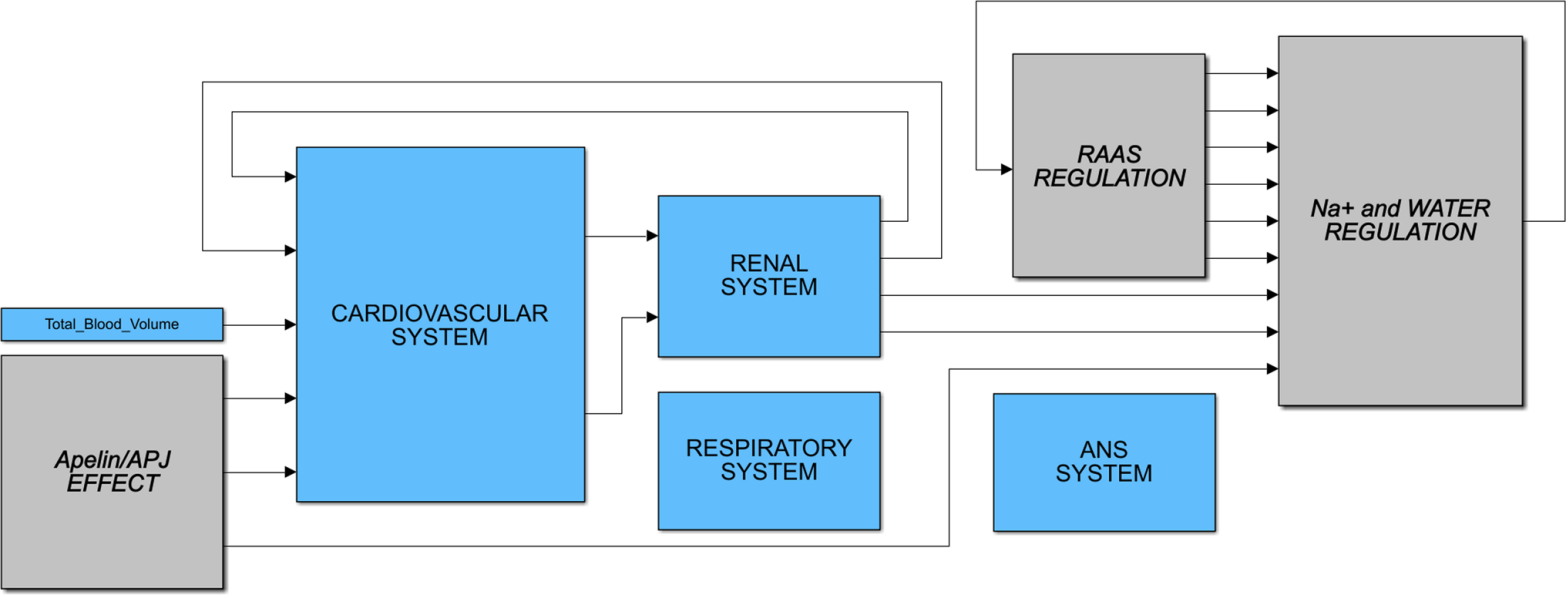
Diagram of a quantitative systems pharmacology model of heart failure

A summary diagram of a QSP model being applied to inform clinical development is shown in Fig. 1. The model mathematically describes the physiology of cardiovascular, respiratory, renal system, Na+/water regulation, and the central nervous and hormonal regulation system. The model can simulate a pulsatile heart and hemodynamics with interactions of neuro-hormonal control and kidney function of a virtual patient as a normal subject or with heart failure. In addition to questions about clinical dosing strategies, the heart failure model has been applied to generate a deeper biological and clinical understanding of the disease states in heart failure. The model has also been used to investigate the mechanisms of action for therapeutic targets in clinical development. The heart failure QSP modeling approaches integrate clinical and non-clinical data and current knowledge in a quantitative and mechanistic fashion to generate actionable predictions.

QSP models can simulate virtual patients (VPs) and one major application of the heart failure QSP modeling is also HF VP simulation. A variety of criteria are used to establish that simulated VPs are biologically plausible, or valid. Biomarkers of plausible VPs should be clinically observed physiological ranges, for example observed ranges of cardiac ejection fraction, left ventricle volume, total peripheral resistance, heart rate, cardiac output, diastolic and systolic pressure, respiration rate, and hormonal concentrations. Plausible VPs can provide prediction results and simulated data to begin to address the challenges for HF drug development. However, with observed clinical biomarker and response data, a set of plausible VPs can also be expanded and developed into a virtual patient population that quantitatively matches multiple summary statistics or distributions of the clinical observations [53–55]. These virtual populations can be used in silico trials to aid in a number of clinical development decisions with projections of anticipated population and trial variability. These insights including outcome distributions and quantitative population differences in the underlying mechanisms can be used for a more quantitative projection of trial outcomes when assessing doses, deciding how to stratify patients for therapy by their biomarkers, analyzing the contribution of components, and making decisions about combination therapy approaches, and deciding to advance or halt clinical development [56].

Another application of heart failure QSP modeling is predicting the progression of HF. Even if the HF patients are considered clinically stable when they are receiving treatment and show no physical signs and symptoms of worsening HF, HF treatments generally do not protect against progressive deterioration of cardiac function. The heart failure QSP model captures the progressive change and status of an HF patient with treatment or without treatment over time.

In addition to the QSP applications introduced, significant work has been done developing computational models of cardiac electrophysiology, cardiomyocytes and mechanics for guiding pharmaceutical therapy and deployment of devices, screening cardiac toxicity, and addressing heart failure questions [57]. It is also worth noting that HF can modulate organ blood flows that are important for tissue concentrations and clearance rates, and physiologically based pharmacokinetic (PBPK) models that model the effective clearance of HF drugs with the changes in organ perfusion associated with HF have been used to assess dosing as associated with the disease state [58, 59]. Extension of PBPK models with simple pharmacodynamic (PD) models to create PBPK-PD models is one additional strategy to predict effects [60], and in addition modeling of cardiac tissue concentrations may further help to predict and assess cardiotoxicity [61]. While QSP approaches have also explicitly included distribution of a drug to target tissues, target binding, and mechanistic pharmacodynamic effects, in some cases it has also been useful to instead assess the impact of therapies through a direct in silico modulation of the target [50].

### Challenges and future directions for QSP modeling in heart failure

HF is a widespread disease and poses a substantial medical need that is only partially being met. Overall, there are more than 26 million HF patients worldwide and more than half of them are 65 years or older. Half of HF patients die in 5 years, and a half of HF patients are re-hospitalized in 6 months. Currently, multiple assessments and tests are used for diagnosis, and half of the patients have no approved treatments. HF research is also expensive: a typical cardiovascular clinical trial can cost $72 million, and half of phase III trials have failed. In addition, research in HF can take 30 years or even longer. As mentioned previously, heart failure often involves multiple organs and systems, results in many complications, and the relevant mechanisms for a patient may not be fully elucidated. Although there is already much clinical data related to HF, important readouts of clinical cardiac function and disease state such as ejection fraction, heart rate, and blood pressure may not be reported and generally are not available in a source electronic format where relationships can be more readily assessed. Clinical research in heart failure is challenging and investment in heart failure has declined [62].

Mechanistic cardiovascular computational modeling has been developed over several decades. For example, blood vessel hemodynamic models were published in 1959 [63], electrophysiological cardiac models were published in 1962 [64], electromechanical models of heart have been published since 1974 [65, 66], detailed computational biology of the heart from structure to function have been published since 1990s [67], and integrated cardiovascular models have been developed since 2000s [68, 69]. However, due to these challenges in HF, relatively few multi-scale mechanistic models that incorporate the diverse pathophysiology from multiple length and time scales have been developed. For example, the QSP model of HF presented here captures multiple temporal scales, from modeling action potential at the time scale of milliseconds to modeling changes in cardiac out over years. For maximal mechanistic insight and ability to explore more therapies, biomarkers, and functions, HF models can in theory also utilize multiple physiological scales, from molecular interactions such as target engagement, signaling pathways [70], molecular cardiomyocyte processes including cross-bridge cycling [51], cardiac tissue remodeling [50], up to systemic flow and blood pressure [50]. Current QSP modeling approaches only consider a limited set of components at the molecular through physiological scale. They can provide quantitative predictions as well as hypotheses for HF study, but the limit of resolution of the understanding they provide may be determined in part by the level of mathematical abstraction that mechanisms are incorporated at. Multiscale models of HF including additional processes incorporated explicitly at the molecular, cellular, tissue, and organ levels would therefore help to investigate additional contributing mechanisms and potential biomarkers in silico to deepen the mechanistic understanding of HF. Ultimately, a process of iterative model development, informed by assessment of model behaviors and available data, may help to further advance HF QSP.

## Machine learning in heart failure

### Applications for machine learning in heart failure

The goals of applying ML to medicine include improving the detection and classification of disease, making better predictions, and improving the personalization of medicine [15]. ML has been applied to HF to reduce cost by improving existing diagnostic and treatment support systems [71]. Current HF diagnosis and management rely upon patients’ history, including their physical examination, and both laboratory and imaging data [72]. Learning from existing data, machine learning methods were applied to improve the accuracy and efficiency in predictions in HF diagnosis [73–75], readmission rate [76, 77], mortality rate [78, 79], and hospitalization rate [80]. While conventional statistical models have been used in heart failure, current state-of-art machine learning and deep learning techniques provide more powerful tools boosting the predictive accuracy [81]. A classification model was built by using a support vector machine (SVM) to classify all patients into three groups: the healthy group, the HF-prone group, and the HF group. Large amounts of physical examination records were used to build the ML model, including heart rate variability test, echocardiography test, electrocardiography test, chest radiography test, six minute walk test, and physical test [82]. Individualized treatment and healthy living choices can be suggested for high-risk patients predicted by ML from electronic health records [83]. Unsupervised clustering can identify phenotype groups in heart failure in baseline clinical characteristics, biomarker values, measures of left and right ventricular structure, and function, and the primary outcome occurrence [46, 84, 85]. The classification of phenotypically heterogeneous HF might aid in optimizing the rate of responders to specific therapies. Rather than the regression or classification tasks, machine learning models can also provide significant analysis on HF. For example, random forest methods can analyze the importance of each feature. The left ventricular ejection fraction was successfully identified as the most relevant feature in predicting the survival of patients [86]. Machine learning models were used to analyze heart failure patients, providing various outputs such as an HF severity evaluation, HF-type prediction, as well as a management interface that compares the different patients’ follow-ups [87].

Many technologies can be used to assist the diagnosis of HF, but there is also heterogeneity in clinical diagnoses due to differences between clinicians. Machine learning may help to improve the accuracy of diagnosis. LVEF is a measurement, expressed as a percentage, of how much blood the left ventricle pumps out with each contraction. Empirically, LVEF is an important classifier for HF [88]. However, patients with normal LVEF may have HF, termed HFpEF. The spatiotemporal variations of LV strain rate during rest and exercise can be used to identify patients with HFpEF and to provide an objective diagnostic classification. The analysis of such left ventricular long-axis function data via ML can improve the diagnosis and understanding of HFpEF [73, 74]. Electrocardiogram (ECG) is a non-invasive and simple diagnostic method that may demonstrate changes in congestive HF. However, the changes in ECG signal can also be difficult to detect accurately. Deep learning methods are therefore widely used to improve the accuracy in detecting congestive HF from ECG, where CNN [89] and LSTM [90] have been applied. Echocardiography is another standard tool for HF characterization and management, while it requires a skilled user and interpreter, and the interpretation of echocardiographic images has varying levels of subjectivity and inter-rater reliability [15, 91]. Artificial intelligence (AI) technologies provide new possibilities for echocardiography to generate an accurate, consistent, and automated interpretation of echocardiograms, thus potentially reducing the risk of human error [92]. An ensemble machine-learning model, consisting of support vector machines, random forests, and artificial neural networks, was developed and a majority voting method was used for conclusive predictions based on echocardiographic images [93]. Combining echocardiography data and electronic health records, random forest achieved significantly higher prediction accuracy than logistic regression [94]. Wearable technologies recording cardiac function and machine learning algorithms can assess compensated and decompensated HF states by analyzing the cardiac response to submaximal exercise [95]. Heart sounds could be an economic and efficient method for a daily and home-based monitor for chronic HF. A model combining feature selection from a classic ML model and a downstream deep learning model was able to accurately identify new chronic HF patients through heart sounds [96].

### Challenges and future directions for machine learning in heart failure

With increasingly available data, the accuracy of ML models can be improved in the data-driven approach. Data is an essential component in ML, QSP, and data-driven discovery. In general, a large amount of good quality training data can help to yield good accuracy of ML approaches. Deep learning has the ability for universal approximation to boost the predictive performance when large data sets are available. But challenges exist in machine/deep learning due to the data requirements. First, patient data and biomarkers collected from different sources or with different protocols may have strong variations and noise [15]. How to reduce the variations and align data from multiple sources together in one ML model is critical to the performance of the ML model. Data from different sources, such as different machines and hospitals, may present challenges [15]. Data from diverse and heterogenous sources typically involve different patient groups, inhomogeneous data collection techniques, and unspecified human errors, leading to nonuniform calibration, accuracy, and reliability, etc.

Because of their mechanistic nature, QSP models may help to flag and further check whether data from multiple sources are largely consistent or conflicting. Second, imbalanced data sets with low representation of a class of interest can significantly reduce the prediction accuracy and performance of both ML and QSP models. Tests that directly couple with the majority classes of the imbalanced data may be better predicted, but cases associated with the minority classes may not be accurately predicted. Many techniques on ML models can be applied to better work with imbalanced data [97]. For example, oversampling training data in the minority classes and using special loss function (e.g. focal loss [98]) are two popular approaches to enhance the predictive power of ML models on minority classes. Some analogous strategies may also facilitate QSP model calibration. For example in analogy to the focal loss strategy, statistical methods that ensure infrequent outcomes and biomarker observations associated with the infrequent outcomes are calibrated properly may be employed. Third, missing values in a dataset is a common phenomenon in potential data and biomarkers, especially for large datasets. Patients might skip or forget to take medicine or measurements. Most ML models require data that has the same dimension for individual components. Data imputation to fill out the missing values is necessary to the designs of an ML model to fully utilize all available information from data [99], and imputation strategies may also be useful for datasets for QSP model calibration. On the other hand, data may be limited number due to various reasons, for example if the data collection is expensive or the cases to be studied are rare. The complexity of the ML model needs to be appropriately selected to avoid overfitting. Similar considerations arise in QSP model calibration, and strategies to account for this have included both judicious selection of the number of parameters that are varied in a virtual population as well as model averaging [56].

Model interpretability is also extremely desirable for ML application on HF to reveal underlying mechanisms. However, the trade-off between model accuracy and model interpretability is a main challenge for ML approaches [100]. The integration of data-driven machine learning methods with mechanistic QSP modeling may be a component to overcome this obstacle.

## Machine learning-assisted QSP for heart failure

**Fig. 2:**
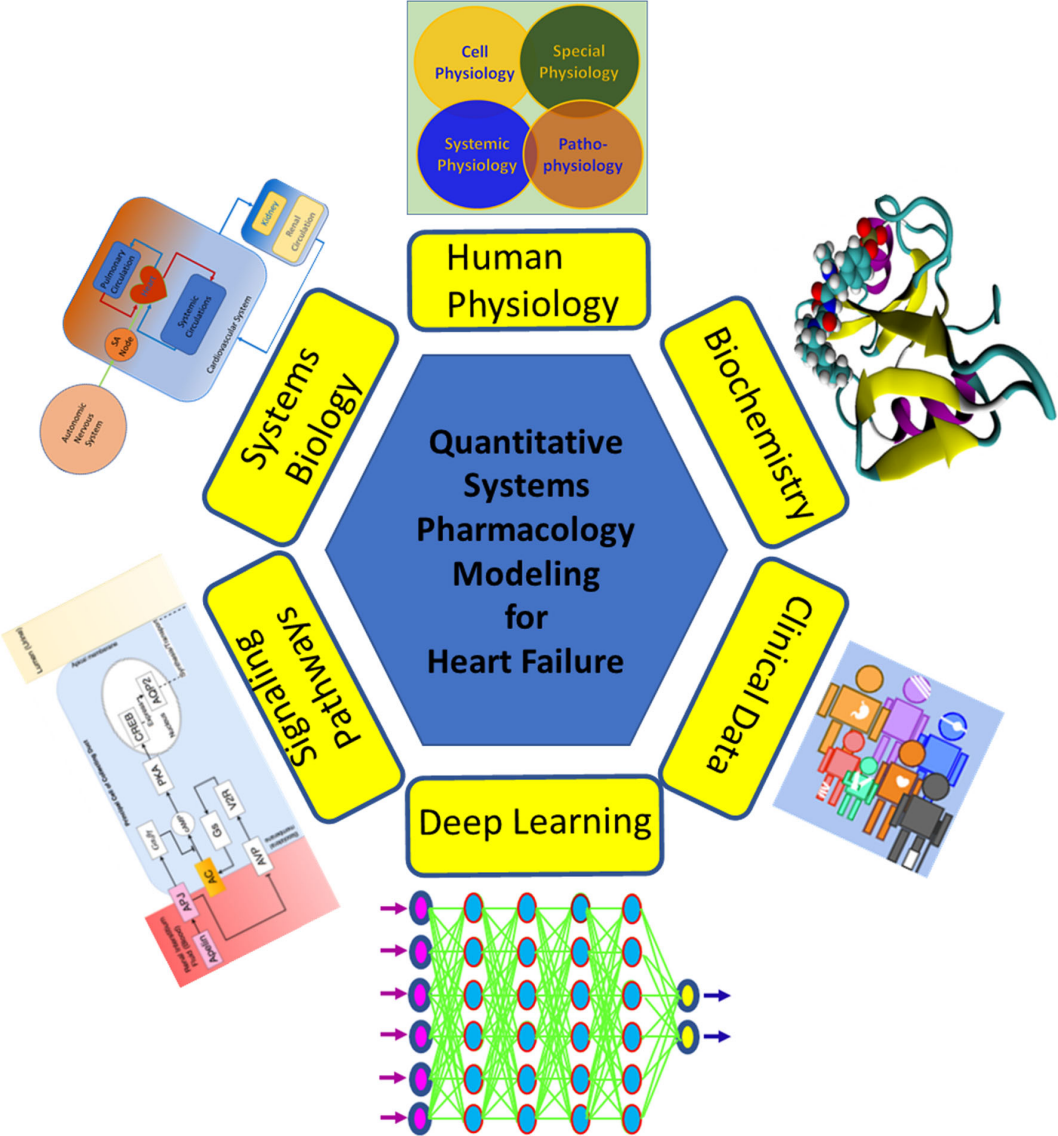
An overview and illustration of machine learning assisted quantitative system pharmacology modeling for heart failure. It involves systems biology, physiology and pathophysiology, biochemistry, signaling pathways, and patient data

In recent years, with the rise of ML and DL technology, QSP modelers have started to apply machine learning-assisted QSP for heart failure modeling (Fig. 2). One key area where we anticipate this will be important is relating clinical trial endpoints that are not mechanistic, such as survival, to endpoints that can be modeled explicitly. For example, we previously described the case where the left ventricular ejection fraction was found to be the most relevant feature in predicting the survival of patients [86]. In this case, the ML training and validation is done explicitly with the clinical data, but the insights can also be used to tie the QSP model outputs to additional clinical information of interest.

A second area where QSP and ML may be integrated is related to the calibration of model event rates using machine learning approaches, once a justifiable link between model outputs and clinical outcomes is set. In certain settings, we are given clinical event rate data reported from many trials with different (drug, dose) combinations, and some of these combinations may be reported from multiple studies with varied outcomes. For each of these studies, we receive the event rate that was reported for the trial. This data can be used to tune an event prediction algorithm. With this strategy, machine learning models are trained at the population level of input data, generated from an ensemble of individual patient QSP simulations. Many ML algorithms can be developed for this purpose. For example, a logistic regression model that takes in the input field(s) for each virtual patient and outputs a probability of an event can be utilized. Other methods, including gradient, boosted decision tree (GBDT), deep neural network (DNN), MDL, can be employed.

A third area where QSP and ML may be integrated is in the development of surrogate models of computationally challenging model subsystems. For example, if some subsystems operate on a relatively fast timescale that imposes smaller time steps on an ordinary differential equation solver, but analytically are not tractable to a good quasi-steady-state approximation, a surrogate model developed with ML that recreates the input-output relationships of interest across all of the conditions to be explored may help to speed model simulations [101]. In our previous study, we used a simple Gaussian regression method as a surrogate for a signaling pathway saturation and desaturation mechanism before calibrating a QSP model of heart failure to publicly available clinical data for a competitor compound, then predicted the potential efficacy of our compound for decision making in the clinical development.

The application of mechanistic models often requires exploration of a high dimensional parameter space, and data-guided methods have been developed to both enable efficient characterization of this space and facilitate virtual population development [56, 102–105]. Highly efficient parameter exploration and model calibration strategies are a fourth area where ML strategies may further assist QSP. Recently, generative models, such as autoencoders, normalizing flows, and generative adversarial network [106], have been explored as a component of new strategies for statistical inference with mechanistic models, and also offer alternatives when the likelihood function is not analytically tractable [107]. Physics-informed neural networks (PINNs) can easily infer parameters and obtain accurate solution of the system with well-known governing Eqs. [108, 109]. PINNs have been applied to cardiac modeling [110]. Moreover, machine learning methods have been also applied to cardiac modeling for uncertainty quantification [111], model order reduction [112], surrogate generation and acceleration of large scale of simulations [113].

## Conclusions

Although, to our knowledge, a QSP model that captures the full range of HF etiologies in good mechanistic detail that explicitly reproduces all proposed contributing factors across the molecular, cellular, tissue, and organ scales has not been developed, we can further develop current HF QSP models to work towards the goal of models capable of reproducing more clinical phenotypes, underlying causes, and calibration to more clinical interventions. We anticipate the utilization of ML approaches will help the advance towards this goal. ML will further bring together different approaches. QSP models that build in with new mechanisms and incorporate richer data will have a bright future.
